# Plasma microRNA levels in male and female children with cystic fibrosis

**DOI:** 10.1038/s41598-020-57964-1

**Published:** 2020-01-24

**Authors:** C. Mooney, P. J. McKiernan, R. Raoof, D. C. Henshall, B. Linnane, P. McNally, A. M. A. Glasgow, C. M. Greene

**Affiliations:** 1 Department of Physiology and Medical Physics, Royal College of Surgeons in Ireland, Dublin, Ireland; 2 School of Computer Science, University College Dublin, Dublin, Ireland; 3 Lung Biology Group, Department of Clinical Microbiology, Royal College of Surgeons in Ireland, Dublin, Ireland; 4 Department of Anatomy, College of Medicine, University of Mosul, Mosul, Iraq; 5 FutureNeuro Research Centre, RCSI, Dublin, Ireland; 6 Study for Host Infection in Early Lung Disease in CF (SHIELD CF), National Children’s Research Centre, Children’s Health Ireland at Crumlin, Dublin, Ireland; 7 Graduate Entry Medical School and Centre for Interventions in Infection, Inflammation & Immunity (4i), University of Limerick, Limerick, Ireland; 8 Department of Paediatrics, Royal College of Surgeons in Ireland, Dublin, Ireland

**Keywords:** miRNAs, Prognostic markers, Cystic fibrosis

## Abstract

A gender gap exists in cystic fibrosis (CF). Here we investigate whether plasma microRNA expression profiles differ between the sexes in CF children. MicroRNA expression was quantified in paediatric CF plasma (n = 12; six females; Age range:1–6; Median Age: 3; 9 p.Phe508del homo- or heterozygotes) using TaqMan OpenArray Human miRNA Panels. Principal component analysis indicated differences in male versus female miRNA profiles. The miRNA array analysis revealed two miRNAs which were significantly increased in the female samples (miR-885-5p; fold change (FC):5.07, adjusted *p* value: 0.026 and miR-193a-5p; FC:2.6, adjusted *p* value: 0.031), although only miR-885-5p was validated as increased in females using specific qPCR assay (*p* < 0.0001). Gene ontology analysis of miR-885-5p validated targets identified cell migration, motility and fibrosis as processes potentially affected, with RAC1-mediated signalling featuring significantly. There is a significant increase in miR-885-5p in plasma of females versus males with CF under six years of age.

## Introduction

Cystic fibrosis (CF) is an autosomal recessive genetic disorder arising from mutations in the cystic fibrosis transmembrane conductance regulator (CFTR) gene^1^. CFTR is a crucial chloride ion channel expressed by epithelial cells in the lung and other organs. The consequences of defective CFTR are most severely manifested in the lung, where the imbalanced ion homeostasis results in chronic airway inflammation, which leads to a progressive and ultimately fatal decline in lung function.

Despite improved therapies and increased life expectancy for both males and females with CF in recent years, a gender dichotomy persists whereby females are at a clinical disadvantage e.g. poorer lung function, earlier colonisation by chronic mucoid *Pseudomonas aeruginosa*, greater frequency of exacerbations, and lower median survival age^2–7^. Whether sex differences exist in children with CF remains controversial^8,9^.

The ability to measure and monitor lung disease in children via a non-invasive method would be highly beneficial for CF clinical care. Molecular biomarkers that are prognostic for lung disease progression could serve, in support of clinical examination, to allow earlier and hence more effective intervention strategies. Ideally these would be molecules that are stable in body fluids (e.g. plasma), able to be quantified with reliance and accuracy, and mechanistically linked to pulmonary infection and/or inflammation.

MicroRNAs are small non-coding RNAs that bind specific target mRNA sequences to inhibit their expression. As well as being expressed within cells/tissues, miRNAs are also present extracellularly, and are readily detectable in body fluids such as serum and plasma. In contrast to mRNA, miRNAs circulating in plasma are surprisingly resistant to RNase degradation due to the fact they are found either packaged inside microvesicles e.g. exosomes, or associated with protein/lipid complexes e.g. argonaute 2 or high density lipoprotein^10^. Although the role of circulating miRNAs is not yet fully understood, their major function is thought to be cell-cell communication, for example between immune cells^10^. It has been demonstrated that circulating miRNAs can be taken up by recipient cells and subsequently cause changes in target gene expression^11,12^. Changes in circulating miRNA expression profiles have been found to correlate with specific disease states, e.g. various cancers, highlighting their potential use as biomarkers^13^.

Many miRNAs have been shown to be dysregulated in the CF lung environment, including those with targets such as CFTR, IL-8, Target of Myb1 (TOM1) and activating transcription factor 6 (ATF6), as reviewed by Glasgow *et al*.^14^. In this work, we sought to explore the circulating miRNA profile of paediatric CF plasma. Given the sex-related differences noted in CF progression, we decided to examine the miRNA profile of samples from males versus females with the disease.

Overall, the aims of this study were to measure miRNAs in plasma from children with CF and to correlate miRNA profiles or differentially expressed (DE) miRNAs with sex, CFTR genotype, age, lung inflammatory markers (neutrophil elastase and interleukin-8), lung infection status and antibiotic treatment.

## Results

### microRNA expression profiles - Over 100 miRNA can be reliably quantified in paediatric CF plasma samples

Analysis of 6 female and 6 male paediatric CF plasma samples was carried out using the QuantStudio 12 K Flex Real-Time PCR System. An average of 157 miRNA with a Ct < 25 were detected per sample before filtering (range: 133–192) (Supp. Fig. 1A). There was no significant difference between the numbers of miRNAs identified in the samples from females versus males. After filtering, 118 miRNA remained (Supp. Fig. 1B) which compares favourably with other reports of miRNA profiles in serum or plasma in healthy adults^15–20^. These miRNA were cross-checked with miRNAs previously identified in 6 other profiling studies of healthy adults (Supp. Table 1). More than half of the miRNA identified are found in at least 4 other studies. Only 5 miRNA were not identified in any of these studies (miR-10b-3p, miR-491-5p, miR-625-3p, miR-636 and miR-7a-1-3p).

The 10 most abundant miRNA here are: hsa-miR-16-5p, hsa-miR-19b-3p, hsa-miR-24-3p, hsa-miR-92a-3p, hsa-miR-223-3p, hsa-miR-191-5p, hsa-miR-150-5p, hsa-miR-29c-3p, mmu-miR-451a and hsa-miR-484. All of these have previously been identified as the most abundant miRNA in other profiling studies of plasma^15^. Potential cell types from which the individual miRNAs could be derived were investigated by comparing to a previous report on miRNA expression profiles in a range of cell types, as shown in Supp. Table 2^21^.

### Correlations between altered miRNA and sex or genotype

**Figure 1:**
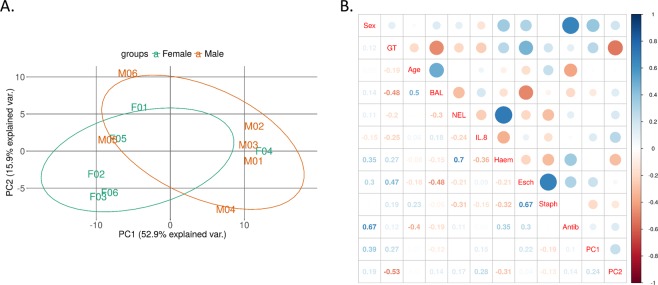
(**A**) Principal component analysis (PCA) was performed on samples after removal of lowly expressed miRNAs (Ct >25) and normalisation of Ct values. (**B**) Correlation plots showing the correlation between sex, genotype (GT), age, BAL Total cell count (BAL), neutrophil elastase levels (NEL), IL-8 concentration (IL-8), *Haemophilus* spp. (Haem), *Escherichia* spp. (Esch), *Staphyloccus* spp. (Staph), antibiotic treatment (Antib), PC1 and PC2. The size of the circles is proportional to the strength of the correlation and the colour represents the direction i.e. a large dark blue circle represents a strong positive correlation and a large dark red circle represents a strong negative correlation.

Next, a principal component analysis (PCA) was performed and the first two principal components (PC1 and PC2) were plotted. This showed some separation of the male and female samples (Fig. 1A). However, this does not fully explain the obvious separation of the samples into two clusters. Despite investigating this in depth, there is no clear explanation for this clustering based on batch effects, age, CFTR genotype, bronchoalveolar lavage (BAL) cell counts, BAL IL-8 or neutrophil elastase levels, infection status or antibiotic treatment. However, ‘Sex’ has the highest correlation with PC1 (r = 0.39) and ‘Genotype’ with PC2 (r = −0.53; Fig. 1B). Upon further investigation, it was apparent there were four miRNAs that correlated highly with PC1: miR-151-3p (r = −0.98), miR-29c-3p (r = −0.74), miR-139-5p (r = 0.72) and miR-132-3p (r = 0.76). Two of these (miR-151-3p and miR-29c-3p) are expressed at very high levels in a number of samples (e.g. miR-151-3p Ct < 10; Supp. Table 3) and drive the data into the two clusters, as demonstrated in Supp. Fig. 2A–D, where hypothetical removal of these two miRNAs removes the clustering of the samples in the PCA.

### miR-885-5p is elevated in plasma from girls versus boys under the age of six with CF

**Figure 2:**
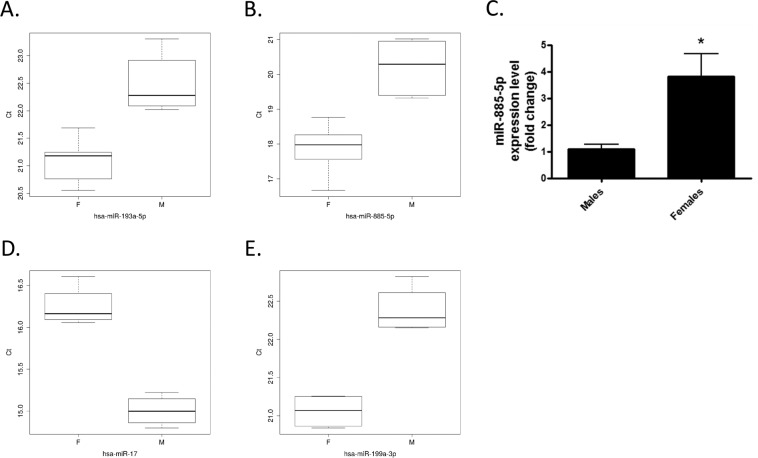
(**A**,**B**) Boxplots showing the distribution of normalised Ct for the two miRNAs found significantly differentially expressed between females and males in the TaqMan OpenArray: (**A**) miR-193a-5p and (**B**) miR-885-5p. (**C**) Validation of differential expression of miR-885-5p by qPCR, *p* < 0.0001. (**D**,**E**) Box plots showing the distribution of normalised Ct for the two miRNAs found differentially expressed between p.Phe508del homozygous females and males in the TaqMan OpenArray: (**D**) miR-17 and (**E**) miR-199a-3p.

The TaqMan OpenArray detected two significantly differentially regulated microRNAs in male versus female samples (Fig. 2A,B); miR-885-5p and miR-193a-5p. Validation for miR-885-5p and miR-193a-5p was performed by individual RT-qPCR on the original 12 CF plasma samples. Whilst miR-193a-5p failed to validate (data not shown), levels of miR-885-5p were confirmed to be significantly elevated in the female versus male CF plasma (Fig. 2C, *p* < 0.0001).

Interestingly, analysis of the p.Phe508del homozygous males versus females (n = 4 per group) revealed significantly decreased levels of miR-17 and increased miR-199a-3p in the female samples (Fig. 2D–E).

### Pathway links between plasma miR-885-5p and pathomechanisms in cystic fibrosis

**Figure 3:**
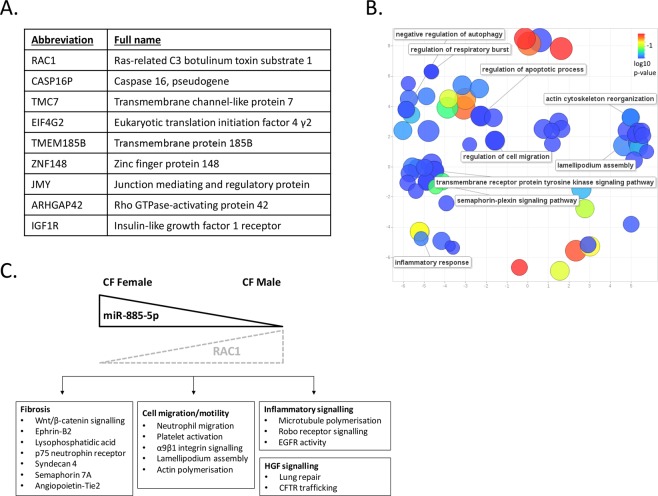
(**A**) Table showing experimentally validated targets of miR-885-5p, as retrieved from miRTarBase. Only targets with evidence from more than one publication are listed. (**B**) Removal of GO term redundancy and semantic visualisation of the significantly associated GO biological processes (adjusted *p* value < 0.05) using REVIGO. The scatterplot shows the cluster representatives (after redundancy reduction) in a two-dimensional space derived by applying multidimensional scaling to a matrix of the GO terms’ semantic similarities (the axes have no intrinsic meaning). Bubble colour indicates the log10 (adjusted *p* value); size indicates the frequency of the GO Biological Process terms in the underlying UniProt database (bubbles of more general terms are larger). (**C**) Schematic summarising RAC1 related pathways by which miR-885-5p may potentially influence CF pathogenesis (dashed lined indicates ‘possible’).

*In silico* analysis of validated targets of miR-885-5p was carried out to gain insight into whether this miRNA may have a pathological role in CF lung disease or the CF gender gap. Experimentally validated targets of miR-885-5p were retrieved from miRTarBase, focusing only on human targets with supporting experimental evidence from more than one publication. Figure 3A shows the nine validated targets of miR-885-5p retrieved from miRTarBase. These were uploaded to Enrichr in order to explore gene ontology biological processes and pathway involvement. The significantly associated gene ontology terms (adjusted *p*-value <0.05) obtained were imported to REGIVO, clustered based on their relatedness, and any redundancy removed. Visualization of these results showed that the largest clusters were formed from the gene ontology biological processes involving tyrosine kinase signalling and cell migration/motility (Fig. 3B). Significantly enriched (adjusted *p*-value <0.001) Reactome, NCI Nature and BioCarta pathways for targets of miR-885-5p obtained from Enrichr are shown in Supp. Tables 4–6. The vast majority of significantly associated gene ontology terms and enriched pathways were associated with RAC1, with the other eight miR-885-5p target genes featuring minimally. A number of potential CF-related pathways were identified including actin cytoskeleton organization, cell migration/motility, microtubule polymerization, Roundabout receptor (Robo) signaling, hepatocyte growth factor (HFG) receptor signaling and fibrosis-related pathways. Pathways of interest are marked by grey shading in Supp. Tables 4–6 and summarized visually in Fig. 3C.

## Discussion

We, and others, have previously shown that numerous miRNAs are differentially expressed in the lungs of CF adults^14,22,23^. The aim of this study was to investigate whether plasma microRNA expression profiles differ between the sexes in children with CF. The main finding of our study is that miR-885-5p is increased in the plasma of female versus male children with CF under the age of six, implicating RAC1-mediated processes as potentially differing between the sexes. To our knowledge, this is the first report of differences in miR-885-5p plasma levels in the context of a lung pathology, however there are previous reports of increased circulating blood levels of miR-885-5p associated with a range of liver pathologies^24^. Extracellular miRNAs can be present in a variety of body fluids due to either active or passive processes and ideally can represent a unique molecular barcode with diagnostic value. The source of the plasma miR-885-5p could not be determined, notwithstanding that the miRNA expression profiles of 18 different cell types were taken into consideration, due to the fact that miR-885-5p does not feature in the top 100 miRNAs expressed in the cell types examined^21^.

Indeed miRNAs have been proposed to hold potential as biomarkers for CF disease progression^25^, as exemplified in a recent study by Krause *et al*. which reported that levels of the Mirc1/Mir17–92 cluster in CF sputum correlated with pulmonary exacerbations, lung function and age^26^. The samples used in that study were predominantly from adults, and correlations with gender were not explored. A previous study by Cook *et al*. on circulating miRNAs in serum found altered expression of three miRNAs between CF patients with liver disease, those without liver disease, and non-CF paediatric controls, as well as a panel of six miRNAs that could distinguish between progressive states of liver fibrosis^27^. Additionally, a recent profiling study comparing plasma miRNAs in CF versus non-CF controls validated the increased expression of miR-486-5p, let-7b and miR-103a-3p in CF plasma^28^. However, neither the serum nor plasma study examined differences between male and female samples. Given that sex differences play a role in CF lung disease progression, in this pilot study the plasma miRNA expression profile of six male versus six female children with CF was compared as a first step towards identifying candidate biomarkers of clinical status that may represent the CF gender gap and/or ultimately be useful for guiding earlier therapeutic interventions. Having identified miR-885-5p as a candidate miRNA that may differentiate male and female CF plasma samples, the biological significance of this alteration needs to be explored in future mechanistic studies. However, based on robust bioinformatic analysis it is possible to speculate on what pathways may be affected.

miR-885-5p has a number of experimentally validated targets (Fig. 3A) and the combined significant gene ontology terms that were retrieved following bioinformatic analysis of these targets pointed to numerous cell processes that are of potential relevance to CF. The majority of the pathways featured RAC1. RAC1 is a member of the Rho family of small (~21 kDa) signalling proteins. Rho GTPases are known to regulate CFTR trafficking and expression^29^, as well as numerous aspects of actin dynamics including actin filament polymerisation. Hepatocyte growth factor (HGF) also featured, which is involved in lung repair by blocking apoptosis in epithelial cells and preventing fibrosis^30^. Together HGF and RAC1 enhance the rescue of CFTR to the cell surface by pharmacological correctors^31^. Importantly, therefore, the observation that miR-885-5p is elevated in female CF plasma may have implications for male versus female responses to CFTR modulator therapy. Lumacaftor is a CFTR corrector that promotes the proper folding and maturation of Phe508del-CFTR whereas Ivacaftor enhances the channel open probability of the mutant CFTR. Lumacaftor alone does not produce changes in CF lung function but does so when applied in combination with Ivacaftor. The underlying mechanism of combined action of these CFTR modulators is to increase Phe508del-CFTR membrane expression to stimulate transepithelial Cl- secretion. Moniz *et al*. reported that restoration of Phe508del-CFTR plasma membrane localization by CFTR correctors can be dramatically improved through stimulation of signalling by RAC1 via HGF^31^. Protein quality control mechanisms remove improperly folded proteins from the plasma membrane and can limit the efficacy of some CFTR modulator drugs, such as Lumacaftor. Activation of RAC1 promotes an interaction between ezrin and NHERF1, and it has been reported that co-exposure of airway cells to RAC1 can triple the efficacy of Lumacaftor^32^. Thus, acute co-treatment with HGF can significantly enhance the chemical correction of Phe508del-CFTR via induction of RAC1. Interestingly, prolonged, 15-day HGF treatment also significantly improves the functional rescue of Phe508del-CFTR by Ivacaftor/Lumacaftor in polarized bronchial epithelial monolayers^33^.

Other common themes among the pathway terms were cell migration/motility, inflammation and fibrosis, each of which are strongly implicated in neutrophil recruitment to the lung and damage to pulmonary tissue.

Interestingly, when the p.Phe508del homozygous individuals were analysed separately (n = 4 per group), significantly decreased levels of miR-17 and increased levels of miR-199a-3p were evident in the female samples, suggesting that genotype-specific effects may indeed exist. miR-17 targets IL-8, and we previously reported decreased levels of miR-17 in CF bronchial brushings^34^, and later suggested how manipulation of miR-17 in CF bronchial epithelial cells could have therapeutic benefit^35^. A recent report by Bardin *et al*. pointed to a role for miR-199a-3p in the chronic inflammation of the CF airway^36^. miR-199a-3p was found downregulated in primary air-liquid interface cultures and bronchial explants from CF versus non-CF controls, and was demonstrated to regulate the expression of inhibitor of nuclear factor kappa-B kinase subunit beta (IKK-β), a signalling intermediary of the NF-κB pathway^36^. No significant difference in expression of miR-199a-3p was found in CF versus non-CF plasma, however the authors have not stated the sex of the patients in each group^36^. Larger cohort studies could confirm whether these miRNAs represent useful clinical biomarkers.

As part of the miRNA profile analysis, filtering conditions were used that removed a miRNA if it had a Ct cycle >25 in more than 80% of samples. PCA analysis of the 118 remaining miRNAs showed a separation of the samples into two distinct clusters. Whilst PC1 showed some correlation with sex and genotype, and PC2 was strongly correlated with genotype, the clustering of the samples was not wholly attributable to any of the subject characteristics recorded here i.e. sex, genotype, lung inflammatory parameters, lung infection status or antibiotic treatment. Instead, the separation appeared to be driven by very high expression levels of two miRNAs: miR-151-3p and miR-29c-3p, although these two miRNAs were not differentially expressed between the sexes. Functionally, miR-151-3p has been shown to affect innate immune signalling by targeting signal transducer and activator of transcription 3 (STAT3) and thereby inhibiting IL-6 production in macrophages^37^. It can also affect cell migration by enhancing E-cadherin expression via the transcription factor Twist-related protein 1 (TWIST1). miR-29c-3p has an important role in the inhibition of apoptosis via targeting of forkhead box O3a (FOXO3A), thereby promoting epithelial cell renewal and preventing pulmonary fibrosis^38^. It also regulates cell migration by targeting cell migration-inducing and hyaluronan-binding protein (CEMIP) and the Wnt/β-catenin and epidermal growth factor receptor (EGFR) signalling pathways^39^. Although highly expressed in CF plasma, interestingly, miR-151-3p and miR-29c-3p are downregulated in CF versus non-CF adult bronchial brushings^22^. More in depth analysis of the functional roles of these miRNAs in CF is warranted.

This was a pilot study, not without limitations, investigating male versus female differences in plasma miRNA expression patterns. The sample groups are small and there is a need to follow up in due course with validation of the findings in a larger sized study. These results warrant a longitudinal study evaluating miRNA expression over time in the same individuals, comparing males versus females, with more clinical outcomes and an expanded cohort in order to fully determine the usefulness of miR-885-5p as a clinical biomarker for monitoring CF disease progression or response to CFTR modulator treatment. We are also interested in examining any potential changes to the miRNA expression profile with the onset of puberty. Our current findings in these paediatric samples are unlikely to be explained by the hormonal influences that are blamed for much of the gender gap in older females, and are thus of even greater interest. Although in depth *in silico* analyses was performed, mechanistic studies were beyond the scope of the current work but are warranted in the future in order to test if increased levels of miR-885-5p in female CF plasma are representative of a functional difference, and therefore potentially useful in monitoring the course of the disease and the CF gender gap. In conclusion, we have recorded significantly elevated levels of miR-885-5p in the plasma of female versus male children with CF, and the data suggest RAC1 may be a key molecule that functions via numerous different pathways, including cell migration and fibrosis, to aggravate CF pathogenesis in females.

## Methods

### Study cohort

Twelve children with CF were recruited (six males; Age range:1–6; Median Age: 3; 8 (66.7%) Phe508del homozygote, 1 (8.3%) Phe508del heterozygote, 2 (16.7%) G551D homozygote, 1 (8.3%) other) (Table 1). Plasma was recovered by blood draw. Bronchoalveolar lavage was performed^40^; total BAL cell counts were recorded and BAL fluid IL-8 and neutrophil elastase levels were measured by ELISA.

**Table 1:** Patient characteristics of the study cohort.

Sex	CFTR Genotype	Age (yr)	BAL cell no. (x10^6^/ml)	NE levels	IL-8 (pg/ml)	*Streptococcus* spp.	*Haemophilus* spp.	*Escherichia* spp.	*Staphylococcus* spp.	*Moraxella* spp.	Antibiotic Treatment
F	p.Phe508del/ p.Phe508del	1	1.2	32.46	185.45	−	−	−	−	−	No
F	p.Phe508del/ p.Phe508del	6	74.7	0.00	192.10	+	−	−	+	−	No
F	G551D/G551D	3	1.1	54.52	276.27	−	+	−	−	−	No
F	G551D/G551D	4	4.2	0.00	1673.19	−	−	−	−	−	No
F	p.Phe508del/ p.Phe508del	1	3.1	0.00	1716.03	-	−	−	−	−	Yes
F	p.Phe508del/ p.Phe508del	2	41	0.00	1745.38	−	−	−	−	−	No
M	p.Phe508del/ p.Phe508del	3	2.7	13454.38	336.85		+	−	−	−	Yes
M	p.Phe508del/ p.Phe508del	4	86	0.00	841.81	−	−	−	−	−	No
M	p.Phe508del/c.2988+1 G > A	2	0.27	0.00	841.81		−	+	+		Yes
M	C489+2 G > T/c.1558 G > T	1	7.5	0.00	109.08		+	−	−		Yes
M	p.Phe508del/ p.Phe508del	3	15	0.00	512.74	+	−	−	−	+	Yes
M	p.Phe508del/ p.Phe508del	3	55.1	227.71	1162.45		+	−	−		Yes

All study participants were recruited as approved by the Ethics Medical Research Committee of Our Lady’s Children’s Hospital Crumlin (GEN/228/11). All acquisition, processing and storage of plasma samples was carried out in accordance with the national guidelines and regulations. Written informed consent was provided by a parent or guardian.

### miRNA profiling

Plasma was obtained from the study participants. Since hemolysis of blood samples can alter the miRNA profile of plasma, only non-hemolysed samples were used in the study. Absorbance at 414 nm was measured by spectrophotometry and samples above 0.25 were excluded due to hemolysis as previously described^15^. RNA was isolated using the miRNeasy Serum/Plasma kit (Qiagen) and reverse transcribed using pre-defined Megaplex™ pool RT-primers. Pre-amplifications were performed and miRNA expression was quantified using TaqMan OpenArray Human miRNA Panels in the QuantStudio™ 12 K Flex Real-Time PCR system, allowing for 754 miRNAs to be quantified per sample.

### Openarray data and statistical analysis

OpenArray profiling data (Gene Expression Omnibus submission GSE132106) were first analysed using the ExpressionSuite Software (Life Technologies). To ensure good quality detection and to avoid false-positives, if the quality control flags “AmpScore” or “CqConf” were <1.24 or <0.8 respectively then the Ct score was set to “Undetermined”. AmpScore and Cq Confidence were provided by ExpressionSuite software. Cut-off thresholds were set in consultation with technical experts at Applied Biosystems.

All further analyses were performed in R/Bioconductor^41^. The data was filtered thereby removing a miRNA if it was “Undetermined” or had a cycle threshold (Ct) score >25 in more than 80% of samples. Note that Ct values are inversely related to expression level i.e. a lower Ct value corresponds to higher expression. Missing data points were imputed (Bioconductor package “Non-detects”^42^) and the data was normalised to the geometric mean (GM) as implemented in Bioconductor package “HTqPCR”^43^. Using these criteria, 636 miRNAs were filtered out, leaving 118 miRNAs in the final data analysis (Fig. 1A,B).

Differential expression analysis was performed by applying a Student’s *t*-test to the normalized Ct values between the two conditions and the *p*-values were adjusted for multiple testing by controlling the false discovery rate (FDR) according to the method of Benjamini and Hochberg^44^. A miRNA was considered to be differentially expressed if the adjusted *p*-value was ≤0.05. Fold changes (FC) were calculated as FC = 2^−∆Ct^, where ∆Ct = Ct_miRNA_ − GM.

The miRNAmeConverter Bioconductor package^45^ was used to convert miRNA names from the miRBase V14 nomenclature, as used on the OpenArray platform, to miRBase V21 nomenclature, as used in the Results and Discussion sections.

### miRNA TaqMan assays

Validation of OpenArray findings was performed using qRT-PCR based TaqMan miRNA assays as recommended by the manufacturer. Mature miRNA expression was measured on a LightCycler® 480. cDNA was thawed on ice and diluted immediately before PCR by adding 200 µl RNase-free H_2_O to the 20 µl cDNA sample. Using the 2 × QuantiTect TaqMan PCR Master Mix, reagents for a 25 µl reaction, including diluted cDNA were mixed and added to each well. Data analysis was performed on Ct values and relative expression was calculated using the 2^−ΔΔCt^ method using U6 small RNA controls. Data were analysed with GraphPad Prism 4.0 software package (GraphPad Software, San Diego, CA). Specific analyses that were performed are described for each figure. Differences were considered significant at *p* < 0.05.

### Pathway analyses and bioinformatics

Experimentally validated microRNA targets for miRNA were retrieved from miRTarBase^46^. Only human targets with evidence from more than one publication were retained. The genes identified were uploaded to Enrichr (gene set enrichment analysis web server)^47^ and the gene ontology biological processes, molecular function and pathway involvement of the targets were explored. The significantly associated gene ontology terms (adjusted *p*-value <0.05) were imported to REVIGO where they were clustered based on their relatedness and any redundancy was removed^48^. Significantly enriched Reactome, NCI Nature and BioCarta pathways with adjusted *p*-value <0.001 were exported from Enrichr to Excel and examined for CF-related processes.

## Supplementary information

Supp Figs 1–2 and Supp Tables 1–3.

Supp Table 4.

Supp Table 5.

Supp Table 6.

## Data Availability

The dataset generated and analysed from the miRNA profiling is publically available at Gene Expression Omnibus (GEO), submission number GSE132106.
