# Supplementary material for: Plasma microRNA levels in male and female children with cystic fibrosis
Source: Sci Rep. 2020 Jan 24;10:1141. doi: 10.1038/s41598-020-57964-1 (PMC6981182; doi:10.1038/s41598-020-57964-1)
Supplement: Supplementary file 1 — Supp Figs 1–2 and Supp Tables 1–3. [file 41598_2020_57964_MOESM1_ESM.pdf]

SUPPLEMENTARY FILE:

Plasma microRNA levels in male and female children with cystic fibrosis

Mooney C[1,2#], McKiernan PJ[3#], Raoof R[1,4], Henshall DC[1,5], Linnane B[6,7], McNally P[6,8], Glasgow AMA[3*], Greene CM[3]

[1]Department of Physiology and Medical Physics, Royal College of Surgeons in Ireland, Dublin, Ireland,

[2]School of Computer Science, University College Dublin,

[3]Lung Biology Group, Department of Clinical Microbiology, Royal College of Surgeons in Ireland,

[4]Department of Anatomy, College of Medicine, University of Mosul, Mosul, Iraq

[5]FutureNeuro Research Centre, RCSI, Dublin, Ireland

[6]Study for Host Infection in Early Lung Disease in CF (SHIELD CF), National Children's Research Centre, Children's Health Ireland at Crumlin, Dublin, Ireland,

[7]Graduate Entry Medical School and Centre for Interventions in Infection, Inflammation & Immunity (4i), University of Limerick, Limerick, Ireland,

[8]Department of Paediatrics, Royal College of Surgeons in Ireland, Dublin, Ireland

[#]Both authors contributed equally to this work.

***Correspondence should be addressed to:**

Dr Arlene Glasgow, Lung Biology Group, Department of Clinical Microbiology, Royal College of Surgeons in Ireland Education and Research Centre, Beaumont Hospital, Dublin 9, Ireland, email: arleneglasgow@rcsi.ie, Tel: (+353) 809-3801

Supplementary Figures:

Supp. Figure 1

A.

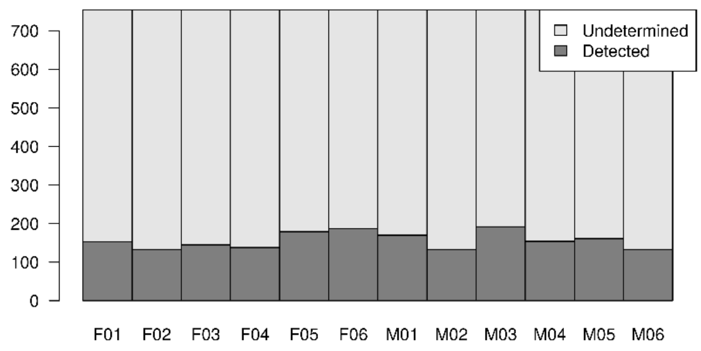

B.

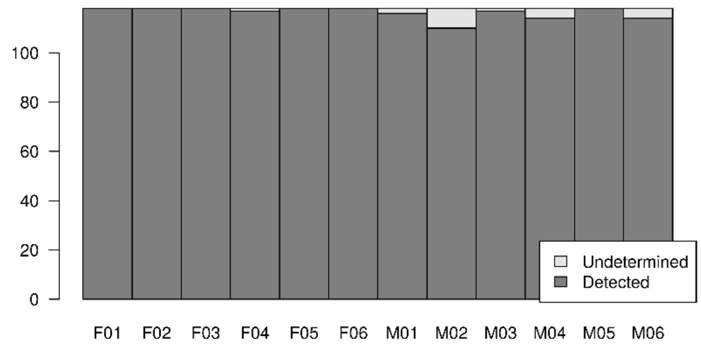

Supp. Figure 1. Bar plot showing the number of microRNA detected in each sample (A) before and (B) after filtering to remove any microRNA with a Ct score > 25 in at least 80% of samples.

Supp. Figure 2.

A.

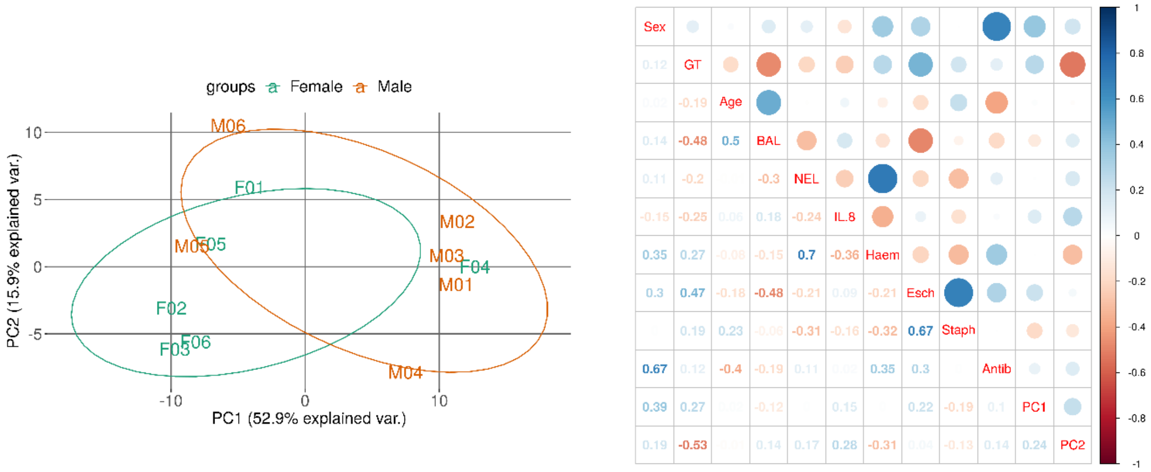

B.

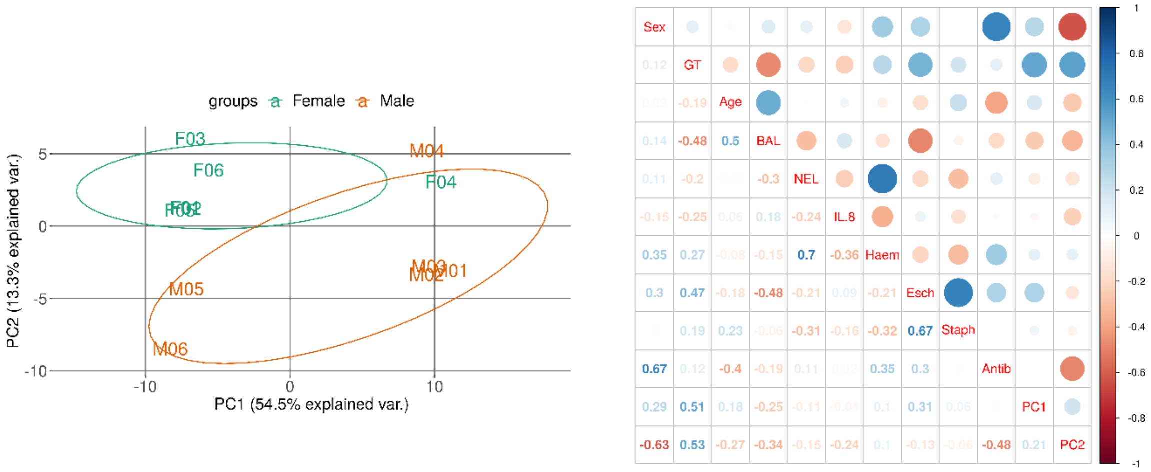

C.

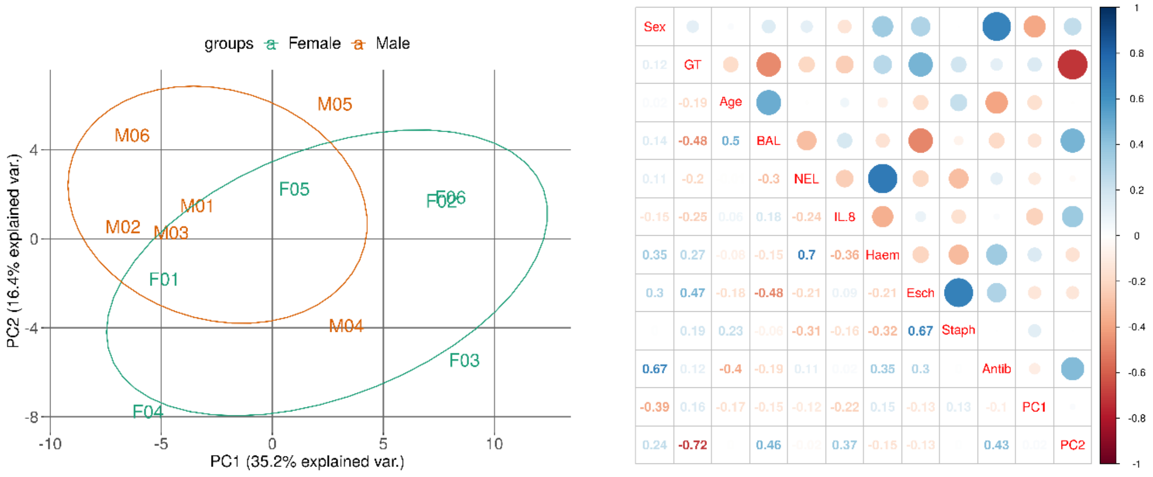
groups Female Male
M05
M06
F05
F06
F02
M01
M02
M03
F01
M04
F03
F04
PC2 (16.4% explained var.)
PC1 (35.2% explained var.)
Sex
GT
Age
BAL
NEL
IL.8
Haem
Esch
Staph
Antib
PC1
PC2

D.

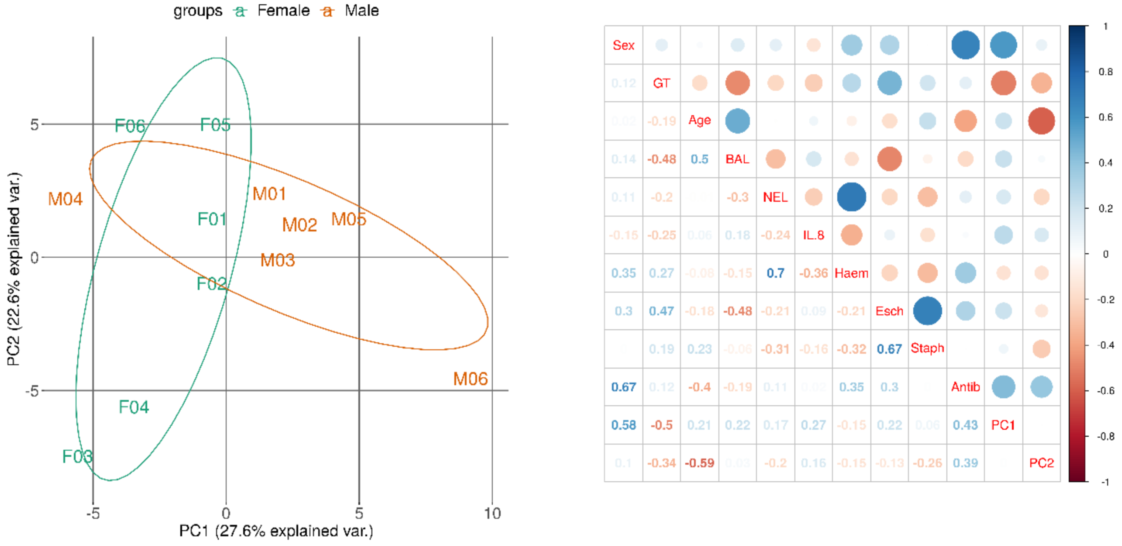
groups Female Male
F06
F05
M04
M01
F01
M02
M05
M03
F02
M06
F04
F03
PC2 (22.6% explained var.)
PC1 (27.6% explained var.)
Sex
GT
Age
BAL
NEL
IL.8
Haem
Esch
Staph
Antib
PC1
PC2

Supp. Figure 2.

(A) All miRNA included in Principal Component Analysis. Principal component 1 (PC1) explains 52.9% of the total variance. Two clusters are clearly visible in the plot which are primarily defined by PC1: M06, F01, M05, F05, F02, F06 and F03; M02, M03, F04, M01 and M04. Sex has the highest correlation with PC1 (r = 0.39) and genotype with PC2 (r = -0.53).

(B) miR-29c alone has been removed from the analysis. PC1 now explains 54.5% of the total variance. Although the same two clusters are seen as before separating along PC1, we can now also see that the samples are separating by sex on PC2. Sex is now strongly correlated with PC2 (r = -0.63) and antibiotic treatment (r = 0.67), and PC1 is correlated with genotype (r = 0.51).

(C) Removing miR-151 alone decreases the separation of the two clusters across PC1 (PC1 now explains 35.2% of the total variance) and strengthens the correlation of PC2 with genotype (r = -0.72).

(D) Both miR-29c and miR-151 have been removed from the analysis. The percentage of the total variance explained by PC1 has dropped from 52.9% to 27.6%; the two clusters are gone. There is a clearer clustering by sex, apart from sample M04. PC1 is correlated with sex, genotype and antibiotic treatment (r = 0.58, -0.5 and 0.43 respectively). PC2 is correlated with age (r = -0.59).

Supplementary Tables:

Supp. Table 1. miRNA identified in 80% of samples and their overlap with miRNA identified in six other studies which profiled plasma or serum in healthy adults. (Only 115 of the 118 miRNA identified are shown as three miRNA were not found in miRBase V21.)

**miRNA**	**Mooney et al 2015**	**Wang et al 2012**	**Blondal et al 2013**	**Weber et al 2010**	**Chen et al 2008**	**Mitchell et al 2008**
miR-106b-5p	*	*	*	*	*	*
miR-17-5p	*	*	*	*	*	*
miR-18a-5p	*	*	*	*	*	*
miR-191-5p	*	*	*	*	*	*
miR-192-5p	*	*	*	*	*	*
miR-19a-3p	*	*	*	*	*	*
miR-20b-5p	*	*	*	*	*	*
miR-21-5p	*	*	*	*	*	*
miR-223-3p	*	*	*	*	*	*
miR-24-3p	*	*	*	*	*	*
miR-25-3p	*	*	*	*	*	*
miR-27b-3p	*	*	*	*	*	*
miR-30b-5p	*	*	*	*	*	*
miR-30c-5p	*	*	*	*	*	*
miR-451a	*	*	*	*	*	*
miR-93-5p	*	*	*	*	*	*
let-7d-5p	*		*	*	*	*
miR-103a-3p	*		*	*	*	*
miR-106a-5p	*	*	*	*	*	
miR-126-3p	*	*	*		*	*
miR-142-3p	*	*	*		*	*
miR-146a-5p	*	*	*		*	*
miR-148a-3p	*		*	*	*	*
miR-16-5p	*	*	*		*	*
miR-186-5p	*	*	*		*	*
miR-195-5p	*	*	*	*		*
miR-199a-3p	*	*	*	*	*	
miR-19b-3p	*	*	*		*	*
miR-20a-5p	*	*	*		*	*
miR-210-3p		*	*	*	*	*
miR-221-3p	*	*	*	*		*

miRNA (contd)	Mooney et al 2015 (contd)	Wang et al 2012 (contd)	Blondal et al 2013 (contd)	Weber et al 2010 (contd)	Chen et al 2008 (contd)	Mitchell et al 2008 (contd)
miR-222-3p	*	*	*		*	*
miR-27a-3p	*	*	*	*		*
miR-29a-3p	*	*	*		*	*
miR-29c-3p		*	*	*	*	*
miR-301a-3p	*	*	*	*	*	
miR-30d-5p	*		*	*	*	*
miR-324-3p	*	*	*	*		*
miR-328-3p	*	*	*	*		*
miR-331-3p	*	*	*	*	*	
miR-484		*	*	*	*	*
miR-532-5p	*	*	*	*	*	
miR-652-3p	*	*	*	*	*	
miR-92a-3p	*	*	*	*	*	
miR-130a-3p	*		*		*	*
miR-133a-3p	*	*	*	*		
miR-15b-5p	*		*		*	*
miR-26b-5p	*		*		*	*
miR-28-3p	*	*	*	*		
miR-324-5p	*	*	*		*	
miR-335-5p	*		*	*		*
miR-345-5p	*	*		*		*
miR-374a-5p	*	*		*	*	
miR-375	*	*	*			*
miR-376a-3p	*	*		*		*
miR-532-3p	*	*	*	*		
miR-590-5p	*	*		*	*	
miR-660-5p		*		*	*	*
miR-99b-5p	*	*	*			*
let-7c-5p	*				*	*
miR-122-5p	*	*	*			
miR-139-5p	*	*	*			
miR-140-3p		*	*		*	
miR-140-5p	*	*	*			
miR-143-3p	*		*	*		
miR-146b-5p	*	*			*	
miR-150-5p	*	*	*			
miR-155-5p	*	*				*
miR-181a-5p	*		*		*	
miR-185-5p		*	*		*	
miR-193a-5p	*	*		*		

miRNA (contd)	Mooney et al 2015 (contd)	Wang et al 2012 (contd)	Blondal et al 2013 (contd)	Weber et al 2010 (contd)	Chen et al 2008 (contd)	Mitchell et al 2008 (contd)
miR-26a-5p	*				*	*
miR-28-5p	*	*	*			
miR-320a	*	*	*			
miR-339-3p	*	*	*			
miR-342-3p		*	*		*	
miR-374b-5p			*	*	*	
miR-410-3p	*			*		*
miR-423-5p	*		*		*	
miR-483-5p	*	*		*		
miR-574-3p	*	*	*			
miR-885-5p	*	*		*		
miR-100-5p				*	*	
miR-125b-5p		*	*			
miR-127-3p	*	*				
miR-132-3p	*			*		
miR-151a-3p				*	*	
miR-193b-3p	*	*				
miR-203a-3p		*		*		
miR-218-5p		*		*		
miR-224-5p				*		*
miR-30a-5p	*			*		
miR-30e-3p	*					*
miR-361-5p	*			*		
miR-376c-3p	*	*				
miR-409-3p	*			*		
miR-454-3p				*	*	
miR-10a-5p		*				
miR-126-5p	*					
miR-139-3p				*		
miR-204-5p				*		
miR-211-5p				*		
miR-214-3p				*		
miR-223-5p	*					
miR-296-5p		*				
miR-30a-3p	*					
miR-365a-3p		*				
miR-766-3p	*					
miR-93-3p	*					
miR-942-5p	*					
miR-10b-3p						

**miRNA (contd)**	**Mooney et al 2015 (contd)**	**Wang et al 2012 (contd)**	**Blondal et al 2013 (contd)**	**Weber et al 2010 (contd)**	**Chen et al 2008 (contd)**	**Mitchell et al 2008 (contd)**
miR-491-5p						
miR-625-3p						
miR-636						
miR-7a-1-3p						

Supp. Table 2. Possible cellular origin of miRNA identified in plasma samples. An asterix is placed in the column if the miRNA is found in the top 100 of miRNA expressed in that cell type. Expression profiles for all cells taken from Haider et al (2014).

	acinar cell	adipocyte	centroblast	ductal cell	endothelial cell	epithelial cell	fibroblast	hepatocyte	lymphatic EC	memory B cell	monocyte	myocyte	naive B cell	neutrophil	NK cell	plasma cell	red blood cell	smooth muscle cell
let-7d-5p	*	*	*	*	*	*	*	*	*	*	*	*	*	*	*	*	*	*
miR-16-5p	*	*	*	*	*	*	*	*	*	*	*	*	*	*	*	*	*	*
miR-17-5p	*	*	*	*	*	*	*	*	*	*	*	*	*		*	*	*	*
miR-18a-5p			*		*			*		*	*		*		*	*	*	
miR-19a-3p	*	*	*	*	*	*	*	*	*	*	*	*	*	*	*	*	*	
miR-19b-3p	*	*	*	*	*	*	*	*	*	*	*	*	*	*	*	*	*	*
miR-20a-5p	*	*	*	*	*	*	*	*	*	*	*	*	*	*	*	*	*	*
miR-21-5p	*	*	*	*	*	*	*	*	*	*	*	*	*	*	*	*	*	*
miR-24-3p	*	*	*	*	*	*	*	*	*	*	*	*	*	*	*	*	*	*
miR-25-3p	*	*	*	*	*	*	*	*	*	*	*	*	*	*	*	*	*	*
miR-26a-5p	*	*	*	*	*	*	*	*	*	*	*	*	*	*	*	*	*	*
miR-26b-5p	*	*	*	*	*	*	*	*	*	*	*	*	*	*	*	*	*	*
miR-27a-3p	*	*	*	*	*	*	*	*	*	*	*	*	*	*	*	*	*	*
miR-28-5p	*	*	*	*	*	*		*		*	*		*			*		*
miR-29a-3p	*	*	*	*	*	*	*	*	*	*	*	*	*	*	*	*	*	*
miR-30a-5p	*	*	*	*	*	*	*	*	*			*				*		*
miR-30a-3p	*				*			*	*									*
miR-92a-3p	*	*	*	*	*	*	*	*	*	*	*		*	*	*	*	*	*
miR-100-5p	*	*	*	*	*	*	*	*	*			*						*
miR-103a-3p	*	*	*	*	*	*	*	*	*	*	*	*	*	*	*	*	*	*
miR-106a-5p																		
miR-140-5p		*	*	*	*		*	*		*	*	*	*	*		*	*	*

	acinar cell	adipocyte	centroblast	ductal cell	endothelial cell	epithelial cell	fibroblast	hepatocyte	lymphatic EC	memory B cell	monocyte	myocyte	naive B cell	neutrophil	NK cell	plasma cell	red blood cell	smooth muscle cell
miR-192-5p	*			*				*			*		*				*	
miR-199a-3p																		
miR-148a-3p	*	*	*	*		*		*		*	*		*	*		*	*	
miR-30c-5p	*		*	*	*	*	*	*	*	*	*	*	*	*	*	*	*	
miR-30d-5p	*	*	*	*	*	*	*	*	*	*	*	*	*		*	*	*	
miR-139-5p									*									
miR-10a-5p	*	*		*	*				*			*			*			
miR-181a-5p	*	*	*	*	*	*	*		*	*	*	*	*	*	*	*	*	*
miR-203a-3p																		
miR-204-5p									*									*
miR-210-3p																		
miR-211-5p														*				
miR-214-3p	*	*		*			*					*		*				*
miR-218-5p				*			*											*
miR-221-3p	*	*	*	*	*	*	*	*	*	*	*	*	*	*	*	*		*
miR-222-3p		*	*	*	*	*	*	*	*	*	*	*	*	*	*	*	*	*
miR-223-3p	*		*	*					*	*	*		*	*	*	*	*	*
miR-224-5p		*		*	*	*		*	*									*
miR-15b-5p	*	*	*	*	*	*	*	*	*	*	*	*	*	*	*	*	*	*
miR-27b-3p	*	*	*	*	*	*	*	*	*		*	*	*		*			*
miR-30b-5p	*	*	*	*	*	*	*	*	*	*	*	*	*	*	*	*	*	
miR-122-5p								*						*				
miR-125b-5p	*	*	*	*	*	*	*	*	*			*			*	*		*
miR-130a-3p	*	*		*	*	*	*	*	*		*	*					*	*
miR-132-3p															*			
miR-133a-3p																		

	acinar cell	adipocyte	centroblast	ductal cell	endothelial cell	epithelial cell	fibroblast	hepatocyte	lymphatic EC	memory B cell	monocyte	myocyte	naive B cell	neutrophil	NK cell	plasma cell	red blood cell	smooth muscle cell
miR-142-3p	*		*	*						*	*		*	*	*	*	*	
miR-143-3p	*	*	*	*	*	*	*	*		*	*	*	*	*	*	*	*	*
miR-191-5p																		
miR-126-5p					*				*		*						*	
miR-126-3p	*			*	*			*	*		*				*		*	*
miR-127-3p		*			*		*		*					*				*
miR-146a-5p			*	*	*				*	*	*		*		*	*		*
miR-150-5p			*	*						*	*		*		*	*		
miR-185-5p			*		*	*		*	*	*	*		*	*	*	*	*	*
miR-186-5p			*		*	*		*	*	*	*		*		*	*	*	
miR-195-5p	*	*	*	*	*			*		*	*	*	*			*	*	*
miR-320a	*	*	*	*	*	*	*	*	*	*	*	*	*		*	*	*	*
miR-93-5p	*	*	*	*	*	*	*	*	*	*	*	*	*	*	*	*	*	*
miR-7a-1-3p																		
miR-155-5p			*		*		*	*	*	*	*		*		*	*		*
miR-106b-5p	*	*	*	*	*	*	*	*	*	*	*	*	*	*	*	*	*	*
miR-29c-3p	*	*	*	*	*	*	*	*	*	*	*	*	*	*	*	*	*	*
miR-301a-3p		*	*			*					*					*	*	
miR-99b-5p		*			*	*	*		*									*
miR-296-5p																	*	*
miR-30e-3p	*		*			*		*		*	*	*	*			*		
miR-361-5p	*	*	*	*	*	*	*	*	*	*	*	*	*		*	*		*
miR-365a-3p	*	*	*	*	*	*	*	*	*	*		*			*	*		*
miR-376c-3p																		
miR-374a-5p	*	*	*	*	*	*	*	*	*	*	*	*	*		*	*	*	
miR-375	*			*				*										

	acinar cell	adipocyte	centroblast	ductal cell	endothelial cell	epithelial cell	fibroblast	hepatocyte	lymphatic EC	memory B cell	monocyte	myocyte	naive B cell	neutrophil	NK cell	plasma cell	red blood cell	smooth muscle cell
miR-376a-3p	*	*		*	*		*		*		*	*						*
miR-328-3p																		
miR-342-3p		*	*	*	*	*	*		*	*	*		*		*	*		
miR-151a-3p			*		*	*	*	*	*	*	*		*			*	*	*
miR-331-3p	*	*	*	*	*	*	*	*	*	*	*	*	*	*	*	*	*	*
miR-324-5p		*	*		*	*	*		*		*	*			*		*	*
miR-324-3p	*	*	*	*	*	*	*	*	*	*	*	*	*	*	*	*	*	*
miR-335-5p	*										*	*						
miR-345-5p									*									
miR-20b-5p	*	*	*	*	*	*		*	*	*	*	*	*		*	*	*	
miR-451a	*			*			*		*	*	*						*	
miR-409-3p					*		*											*
miR-410-3p																		
miR-484			*						*	*	*		*		*	*	*	
miR-146b-5p	*	*		*				*		*	*		*		*	*		
miR-193b-3p	*	*	*		*	*	*	*		*		*	*			*		*
miR-532-5p					*							*					*	
miR-574-3p	*			*			*	*	*	*	*		*	*				
miR-590-5p		*	*			*					*		*				*	
miR-636														*		*		
miR-652-3p										*	*		*	*	*		*	
miR-660-5p					*	*						*	*				*	
miR-491-5p																		
miR-374b-5p																		
miR-454-3p			*								*		*			*	*	
miR-766-3p			*				*			*	*		*	*	*	*	*	*

	acinar cell	adipocyte	centroblast	ductal cell	endothelial cell	epithelial cell	fibroblast	hepatocyte	lymphatic EC	memory B cell	monocyte	myocyte	naive B cell	neutrophil	NK cell	plasma cell	red blood cell	smooth muscle cell
miR-28-3p																		
miR-93-3p																		
miR-139-3p																		
miR-10b-3p																		
miR-223-5p																		
miR-140-3p																		
miR-193a-5p																		
miR-339-3p																		
miR-423-5p																		
miR-483-5p																		
miR-532-3p																		
miR-625-3p																		
miR-885-5p																		
miR-942-5p																		

Supp. Table 3. miRNA expression profiles of 4 miRNA that are highly correlated with PC1 in Fig 2A.

	**miR-151-3p**	**miR-29c-3p**	**miR-139-5p**	**miR-132-3p**
**Cluster 1**				
F01	21.835	9.308	19.234	20.053
F02	21.8565	22.57956	18.69627	19.81074
F03	22.60007	21.80644	19.39069	20.15943
F05	22.15172	14.94329	19.06029	19.86881
F06	21.01779	20.98795	19.53106	19.80603
M05	22.43684	19.51577	19.37146	19.66934
M06	22.63178	11.7722	20.15691	20.08868
**Cluster 2**				
F04	4.779844	7.331018	20.17747	20.27104
M01	5.540158	11.72191	20.09898	20.52778
M02	5.624776	8.976539	19.56518	20.21002
M03	6.115234	10.58235	20.21579	21.06482
M04	6.961962	16.05474	20.18447	20.40124
